# The non-diuretic hypotensive effects of thiazides are enhanced during volume depletion states

**DOI:** 10.1371/journal.pone.0181376

**Published:** 2017-07-18

**Authors:** Saeed Alshahrani, Robert M. Rapoport, Kamyar Zahedi, Min Jiang, Michelle Nieman, Sharon Barone, Andrea L. Meredith, John N. Lorenz, Jack Rubinstein, Manoocher Soleimani

**Affiliations:** 1 Department of Pharmacology and Cell Biophysics, University of Cincinnati, College of Medicine, Cincinnati, OH, United States of America; 2 Center on Genetics of Transport and Epithelial Biology, University of Cincinnati, Cincinnati, OH, United States of America; 3 Division of Nephrology, Department of Medicine, University of Cincinnati, College of Medicine, Cincinnati, OH, United States of America; 4 Research Services, Veterans Affairs Medical Center, Cincinnati, OH, United States of America; 5 Division of Cardiology, Department of Medicine, University of Cincinnati, College of Medicine, Cincinnati, OH, United States of America; 6 Department of Physiology, University of Cincinnati, College of Medicine, Cincinnati, OH, United States of America; 7 Department of Physiology, University of Maryland School of Medicine, Baltimore, Maryland, United States of America; University Medical Center Utrecht, NETHERLANDS

## Abstract

Thiazide derivatives including Hydrochlorothiazide (HCTZ) represent the most common treatment of mild to moderate hypertension. Thiazides initially enhance diuresis via inhibition of the kidney Na^+^-Cl^-^ Cotransporter (NCC). However, chronic volume depletion and diuresis are minimal while lowered blood pressure (BP) is maintained on thiazides. Thus, a vasodilator action of thiazides is proposed, likely via Ca^2+^-activated K^+^ (BK) channels in vascular smooth muscles. This study ascertains the role of volume depletion induced by salt restriction or salt wasting in NCC KO mice on the non-diuretic hypotensive action of HCTZ. HCTZ (20mg/kg s.c.) lowered BP in 1) NCC KO on a salt restricted diet but not with normal diet; 2) in volume depleted but not in volume resuscitated pendrin/NCC dKO mice; the BP reduction occurs without any enhancement in salt excretion or reduction in cardiac output. HCTZ still lowered BP following treatment of NCC KO on salt restricted diet with paxilline (8 mg/kg, *i*.*p*.), a BK channel blocker, and in BK KO and BK/NCC dKO mice on salt restricted diet. In aortic rings from NCC KO mice on normal and low salt diet, HCTZ did not alter and minimally decreased maximal phenylephrine contraction, respectively, while contractile sensitivity remained unchanged. These results demonstrate 1) the non-diuretic hypotensive effects of thiazides are augmented with volume depletion and 2) that the BP reduction is likely the result of HCTZ inhibition of vasoconstriction through a pathway dependent on factors present in vivo, is unrelated to BK channel activation, and involves processes associated with intravascular volume depletion.

## Introduction

Thiazide derivatives are the most commonly used diuretics for the treatment of mild and moderate hypertension [1]. Despite the recognized hypotensive effect of thiazides derivatives such as hydrochlorothiazide (HCTZ), the mechanism by which they reduce the blood pressure (BP) remains uncertain. Originally, the common belief was that thiazides reduce BP primarily via enhancement of salt excretion due to the inhibition of Na^+^-Cl^-^ Co-transporter (NCC) in the kidney distal convoluted tubules (DCT) [2–4]. However, subsequent studies have shown that thiazides may cause a reduction in peripheral vascular resistance [5–7].

Despite the fact that HCTZ hypotensive effects are initiated by fluid excretion and acute plasma volume and cardiac output reduction, chronic treatment of HCTZ however produces less salt excretion followed by plasma volume recovered to the pretreatment levels, while the BP is still reduced [5, 8]. It is not clear whether the diuretic phase; *i*.*e*., intravascular volume depletion is necessary for the following non-diuretics hypotensive effects of HCTZ. Distinguishing the non-diuretic mechanism of thiazides vs. their natriuretic (renal) effect in humans or experimental animals, while very important, is complicated by the fact that both mechanisms may be operating simultaneously, therefore hindering a clear distinction between the two anti-hypertensive modalities. Therefore, these studies aimed to examine the non-diuretic, BP lowering action of HCTZ, utilizing an *in vivo* system, which is lacking the renal target, NCC, and volume depletion due to salt wasting or salt restriction.

In addition to NCC, the Cl-/HCO3- exchanger pendrin (SLC26A4) is predominantly expressed on the apical membrane of non A intercalated cells in the connecting tubules (CNT) and cortical collecting duct (CCD) and plays an important role in the maintenance of salt absorption when NCC is ablated or inhibited [9–11]. Indeed, the ablation or the downregulation of pendrin in the setting of NCC inactivation causes severe salt wasting in pendrin/NCC double KO mice [9].

Studies in *in vitro* systems have shown the ability of thiazides to cause vasodilation in arterial system [6, 7, 12]. The activation of large conductance Ca^+^ activated K^+^ (BK) channels have been suggested to be the mediator of the non-diuretic antihypertensive effects of HCTZ. However, the reports on the universality of thiazide effects on vascular resistance are conflicting. In addition, the clinical significance of such an effect in lowering the BP has not been delineated.

The purposes of these studies were to identify whether volume depletion induced by salt restriction or salt wasting *per se* activates the renin-angiotensin system and whether it exaggerates the non-diuretics BP lowering effects of thiazides. Moreover, we also tested whether the direct vascular action via BK channels are the actual mechanism by which HCTZ reduces the BP independently of its diuretics actions. Toward these goals, mice with the genetic deletion of HCTZ renal target, NCC, were used. Moreover, these mice were subjected to volume depletion by either reducing their dietary salt intake or cross breeding them with pendrin KO mice *i*.*e*., pendrin/NCC double KO (dKO) mice, which exhibit Na^+^ wasting and volume depletion. Systemic BP was monitored by intra-arterial catheter and computerized tail cuff. In addition, cardiac function was examined in response to HCTZ by echocardiography. To investigate the mechanism by which HCTZ reduces the BP, BK channels blocker and BK KO mice were used to test the role of BK channels in HCTZ effects. Vascular reactivity preparation also was used to test the direct action of HCTZ in the aorta vessels of these mice.

## Materials and methods

### Animal husbandry

Studies outlined in this section were designed using ARRIVE guide lines and approved by University of Cincinnati’s Institutional Animal Care and Use Committee (IACUC, protocol number 04020901). All animal handlers were IACUC-trained. Animals had access to food and water ad libitum, were housed in humidity, temperature, and light/dark controlled rooms, and were inspected daily. Animals were euthanized by an over dose (150μl) of Euthazol (390mg Sodium pentobarbital and 50mg phenytoin/100 ml) according to institutional guidelines and approved protocols.

### Experimental animal models and tail DNA genotyping

WT, NCC KO, pendrin/NCC dKO, BK KO, BK/NCC dKO mice were used for these studies. Genotyping was performed as described in the original papers reporting the generation of NCC KO, pendrin KO, and BK KO mice [13–15] respectively. The double BK/NCC KO mice were generated by crossing mice with single deletion of BK or NCC with each other. Generation of double BK/NCC dKO mice was verified by tail genotyping as will be discussed below.

### Generation of BK/NCC dKO mice

NCC KO mice were crossed with BK KO mice. The primers used for genotyping of NCC KO mice were as follows: KO forward, AGG GTC AAG GGC ACG GTT GGC;KO reverse, GGT AAA GGG AGC GGG TCC GAG G;KO reverse phosphoglycerate kinase promoter (pk),GCA TGC TCC AGA CTG CCT TG. The PCR conditions were as follows: initial denaturation, 2 min at 94 °C; followed by annealing, 35 cycles at 94 °C for 30 s; and final extension, 68 °C for 1 min. The amplified DNA samples were examined for the presence of 265-bp (WT) and 188-bp (NCC KO) PCR products.

The primers for BK genotyping were as follows: The first set of BK specific oligos: Neo forward ATA GCC TGA AGA ACG AGA TCA GC; RA14025 reverse CCT CAA GAA GGG GAC TCT AAA C exclusively amplified a 1-kb (BK KO) fragment of the ablated BK gene. The second set of BK oligos: Exon 1 forward TTC ATC ATC TTG CTC TGG CGG ACG 3’; WT reverse CCA TAG TCA CCA ATA GCC C specifically amplified a 332-bp fragment of the WT BK gene. The PCR conditions for both reactions were as follows: initial denaturation for 2 min at 94 °C; followed by annealing, 35 cycles at 94 °C for 30 s; extension, 63 °C for 30 s; and final extension, 68°C for 2 min. Products of the two PCR reactions were size-fractionated. WT and KO mice were identified by the exclusive presence of KO (1 kb) or WT (332 bp) fragments. Heterozygote mice were detected by expressing both fragments. S1 Fig shows a representative tail genotyping PCR gel indicating the identification of BK KO, NCC KO and BK/NCC KO mice.

### Tail cuff measurements

BP measurements were obtained using CODA Non-Invasive BP software (Kent Scientific Corporation, CT, USA) as described [16]. Mice (8–10 weeks of age) were placed on the warming pad until their tails temperature reached 30−32^0^. Systolic arterial pressure was measured and recorded. Accordingly, mice were restrained in special holding tube, and two cuffs placed on their tails. At least 5 consecutive readings were recorded per each animal. Treatment with 20 mg/kg (s.c.) of HCTZ, dissolved in propylene glycol/ethyl alcohol in a 4:1 ratio [17], was repeated twice with at least 2–3 days washout period between treatments, and the average was calculated. This dose was chosen based on published reports, which have utilized HCTZ concentrations that are either similar or higher than the dose in our studies [18, 19]. Two recent reports have used HCTZ at 50 mg/kg [19, 20].

### Intra-arterial BP

The mice were anesthetized with intraperitoneal injections of 50ug/g BW ketamine and 100ug/g BW thiobutabarbital. The mice were placed on a thermally controlled surgical table. The right femoral artery and vein were cannulated with a catheters fashioned from pulling 0.25-in OD and 0.375-in OD polyethylene tubing over a flame. The femoral arterial catheter was connected to a COBE CDXIII pressure transducer to record blood pressure. The femoral vein was used to deliver a maintenance infusion of PBS at a rate of 0.15ul/min/g BW. After baseline measurements were recorded, a subcutaneous injection of either a vehicle or 20mg/kg HCTZ (s.c.) was delivered. Blood pressure and heart rate were recorded at baseline and every five minutes after the injection for up to one hour as described before [21, 22].

### Echocardiography

Echocardiography measurements were taken at baseline and at 5-minute intervals for a total of one hour after HCTZ treatment. All echocardiographic studies were performed as previously described [23]. Briefly, mice were anesthetized with isoflurane and placed on the heated stage of the Vevo 2100. Parasternal long axis (PSLAX) images were recorded and then analyzed on a separate work station with VevoStrain software (Vevo 2100, v1.6, Visualsonic, Toronto, Canada). From the M-mode images, left ventricular cavity size for systole and diastole was measured. The rest of the echocardiographic calculations; Ejection Fraction (%EF), Fractional Shortening (FS), Stroke volume (SV) and Cardiac Output (CO), were calculated using the Vevo software.

### Western blot analysis

Plasma membrane proteins were extracted from mouse kidneys as previously described [10], and were size-fractionated by SDS/PAGE (40μg/lane) and transferred to nitrocellulose membrane. Western blot analyses were performed using anti-renin) antibody (MyBioSourc #315812). Appropriate horseradish peroxidase-conjugated IgGs (Thermo Scientific, Rockford, IL) were used as secondary antibodies. The bands were visualized by chemiluminescence method (Invitrogen, Carlsbad, CA) and captured on light-sensitive imaging film (Denville Scientific Inc, Metuchen, NJ).

### Immunofluorescence labeling studies

Immunolabeling studies were performed as described [24]. Briefly, animals were euthanized with an overdose of pentobarbital sodium and perfused through the left ventricle with 0.9% saline followed by cold 4% paraformaldehyde in 0.1 M sodium phosphate buffer (pH 7.4). Kidneys were removed, cut in tissue blocks, and fixed in formaldehyde solution overnight at 4°C. The tissue was either frozen on dry ice or fixed in paraffin, and 6-μm sections were cut with a cryostat and stored until used. Single-immunofluorescence labeling with renin antibody (MyBioSourc #315812) was performed.

### Vascular reactivity

Contractile experiments were performed as previously described [25, 26]. Experiments with thoracic aorta from NCC KO with normal salt diet and NCC KO with low salt diet were performed in parallel. Aorta from each mouse type was exposed in situ, cleaned of surrounding tissue, and divided into two, ~5 mm duplicate ring segments. Segments were placed in organ baths containing physiologic salt solution (mmole/L: NaCl 118.0, KCl 4.73, NaHCO_3_ 25.0, MgSO_4_ H_2_O 1.15.7, CaCl_2_ 2.5, NaH_2_PO_4_ 1.2, and glucose 5.5; pH 7.4 with 95%O2/5% CO2 bubbling at 37°C). Optimal resting tension of 3 g-force was applied and maintained throughout the experiment. After a 30 min rest period, segments were challenged with test concentrations of KCl (50 mM) and then phenylephrine (3 μM; with cumulative phenylephrine additions or as a single concentration), followed by 15 min exposure to either 100 μM HCTZ or vehicle (DMSO, 0.1%). Phenylephrine was then added cumulatively and, after repeated wash, HCTZ or vehicle added to vessels previously challenged with vehicle and HCTZ, respectively. After 15 min, segments were again challenged with cumulative phenylephrine concentrations. Two of the constrictor responses to 50 mM KCl in aorta from NCC mice exposed to normal and low salt diet were utilized in pervious work [26].

### Statistical analysis

All the results are presented as means ± SE with “n” representing the number of mice in each legend. For two-group comparison, either a paired or unpaired Student's t test was performed, whereas statistical significance between multiple groups was assessed by one-way ANOVA or a two-way with repeated measures as appropriate. If significance was indicted, post-hoc testing was performed using the bonferroni method to compare individual means. The statistical significance for intra-arterial BP in anesthetized mice was determined by one-way ANOVA with repeated measures. In vitro contractile experiments, contraction to phenylephrine was calculated as percent KCl contraction, force in mN/g wet weight, and percent 3 μM phenylephrine contraction. Constrictor responses from the two segments derived from one mouse were averaged. Mean ± SE of the maximal contractile response and EC50’s, expressed as pD2 values, were determined. All statistical analyses were performed using GraphPad Prism 5 analysis software and the significance accepted at p ≤ .05.

## Results

### Vascular volume depletion and effect of angiotensin receptor blockers on BP in pendrin/NCC dKO mice

**Fig 1:**
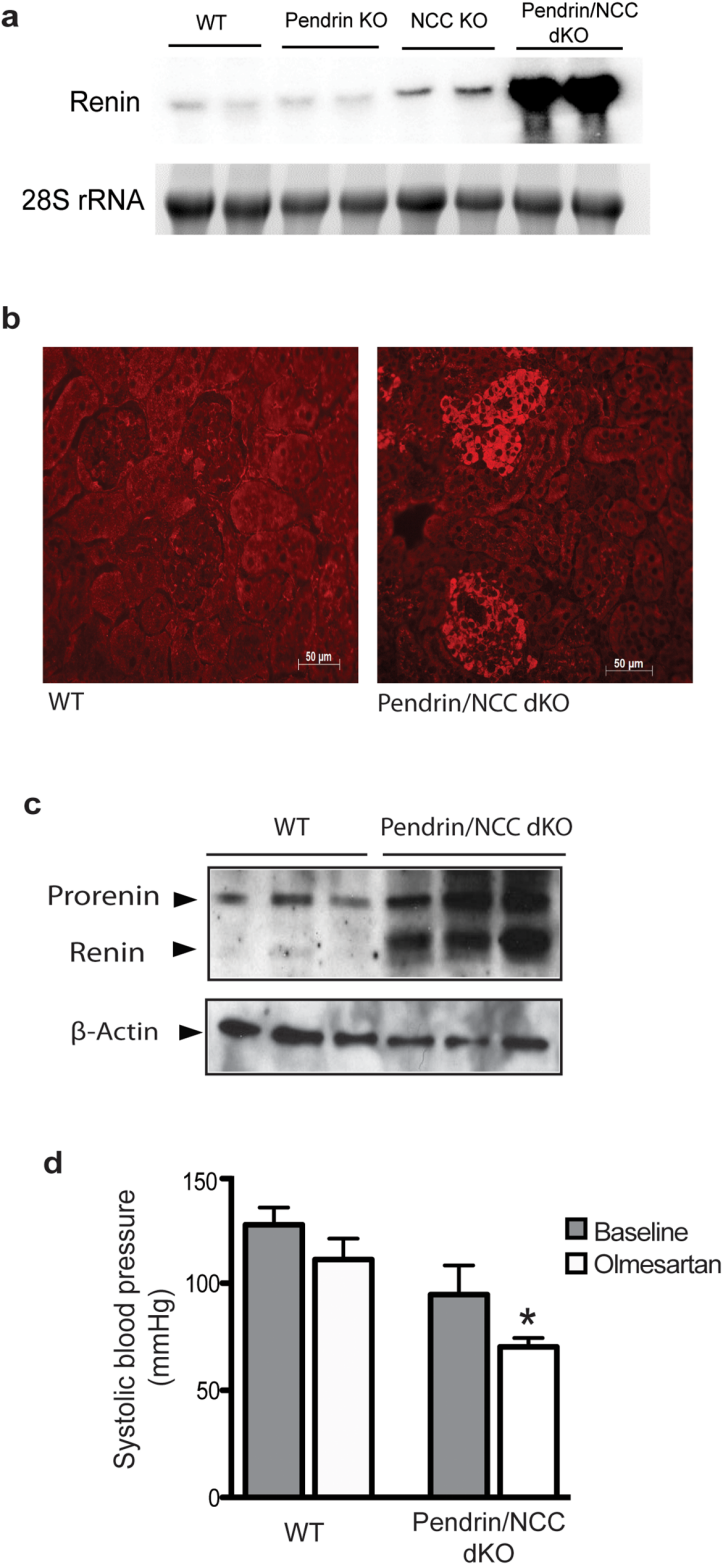
Intravascular volume depletion in Pendrin/NCC dKO mice. (a) Northern hybridization shows a significant enhancement in mRNA expression levels of renin in kidneys of pendrin/NCC dKO mice compared to WT, pendrin KO and NCC KO. (b) Immunofluorescent microscopic analysis of kidney sections indicates that the expression of renin is significantly up regulated in pendrin/NCC dKO, but not in WT mice. (c) Western blots confirmed the significant increase in renin expression in pendrin/NCC dKO kidney vs WT. (d) Olmesartan (1 mg/kg) treatment causes a more significant reduction in the systolic BP of pendrin/NCC dKO mice compared to WT mice, (n = 4 each group); paired t-test * P<0.05.

We first examined the expression of renin in kidneys of WT, pendrin KO, NCC KO and pendrin/NCC dKO mice. As shown in Fig 1A, the expression of renin mRNA was profoundly increased in kidneys of pendrin/NCC dKO mice. Fig 1B represents and immunofluorescence staining and Fig 1C depicts a western blot demonstrating significant enhancement in the expression of renin in kidneys of pendrin/NCC dKO mice vs. WT animals (Fig 1B and 1C). To verify the role of renin angiotensin system activation in blood pressure homeostasis in pendrin/NCC dKO mice, the effect of Olmesartan, an angiotensin receptor blocker, was examined. As shown in Fig 1D, treatment with Olmesartan (1 mg/kg, *s*.*c*.), a potent angiotensin II receptor blocker (ARB), resulted in a significant reduction in systemic blood pressure in pendrin/NCC dKO mice at 1 hour post treatment using a tail-cuff method.

### Effect of HCTZ on systemic BP in WT and pendrin/NCC dKO mice

As shown above, pendrin/NCC dKO mice display salt wasting and volume depletion and develop significant hypotension in response to Olmesarten, an ARB analog [9]. We next tested the effect of HCTZ on systemic BP in WT and pendrin/NCC dKO mice.

**Fig 2:**
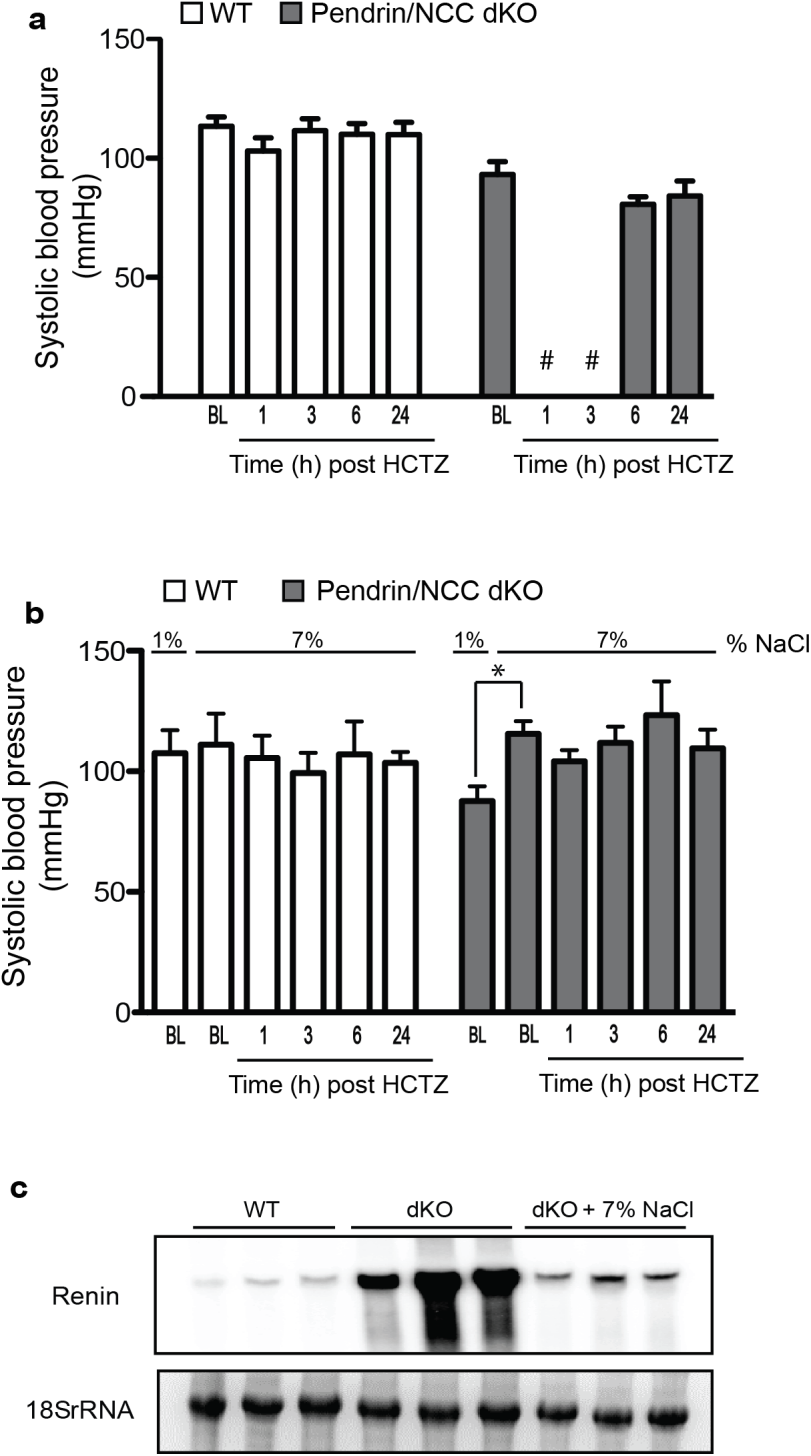
Effects of HCTZ on Pendrin/NCC dKO mice: Role of volume depletion. (a) The systolic BP of pendrin/NCC dKO mice dropped below detectable levels at 1 and 3 hours after HCTZ treatment but returned to baseline levels at 6 and 24 hours later; the systolic BP of WT mice was not significantly affected by HCTZ (#; below detectable level; n = 4). (b) Baseline (BL) blood pressure of pendrin/NCC dKO mice and WT mice was measured on normal (1% NaCl) diet. Then, mice were placed on high salt (7% NaCl) diets for up to 14 days. On day 14, animals’ baseline (BL) blood pressure was measured and then treated with HCTZ, and their BP was measured at 1, 3, 6, and 24 hours (h). The effect of HCTZ is abrogated in euvolemic pendrin/NCC dKO mice. (**c**) Treatment with a high salt diet reduces the renal expression of renin in pendrin/NCC dKO mice, indicating the correction of the volume depletion (n = 3).

Fig 2A depicts BP recordings in WT and pendrin/NCC dKO mice before and at 1, 3, 6, and 24 hrs after (20mg/kg s.c.) HCTZ treatment. As indicated, HCTZ drastically reduces the BP of pendrin/NCC dKO mice while minimally affecting BP of WT animals. The BP in pendrin/NCC dKO mice was reduced below the detectable levels at 1 hour and 3 hours and recovered to near baseline levels by 6 hours after HCTZ treatment (Fig 2A). These findings indicate that HCTZ has a robust hypotensive effect in volume depleted pendrin/NCC dKO mice but not in WT mice.

Fig 2B and 2C examines the role of intravascular volume depletion in the observed lowered BP by HCTZ in pendrin/NCC dKO mice. WT and pendrin/NCC dKO mice were placed on high salt (7% NaCl) diets for up to 14 days; on day 14 animals were treated with HCTZ, and their BP was measured. Fig 2B demonstrates that the effect of HCTZ on BP is abrogated in salt-repleted pendrin/NCC dKO mice. Fig 2C shows that a high salt diet significantly reduces the renal expression of renin in pendrin/NCC dKO mice, consistent with the correction of volume depletion.

To determine whether the hypotensive effect of HCTZ is via enhanced salt excretion, pendrin/NCC dKO animals were placed in metabolic cages for balance studies. As shown in S2 Fig, HCTZ caused a significant reduction in urine output, as well as water and food intake in pendrin/NCC dKO compared to WT mice. The unexpected reduction in food and water intake and urine output in response to HCTZ is likely due to a reduction in systemic BP and hypoperfusion of various organs, including kidneys.

### Effect of HCTZ on the intra-arterial BP in anesthetized mice

**Fig 3:**
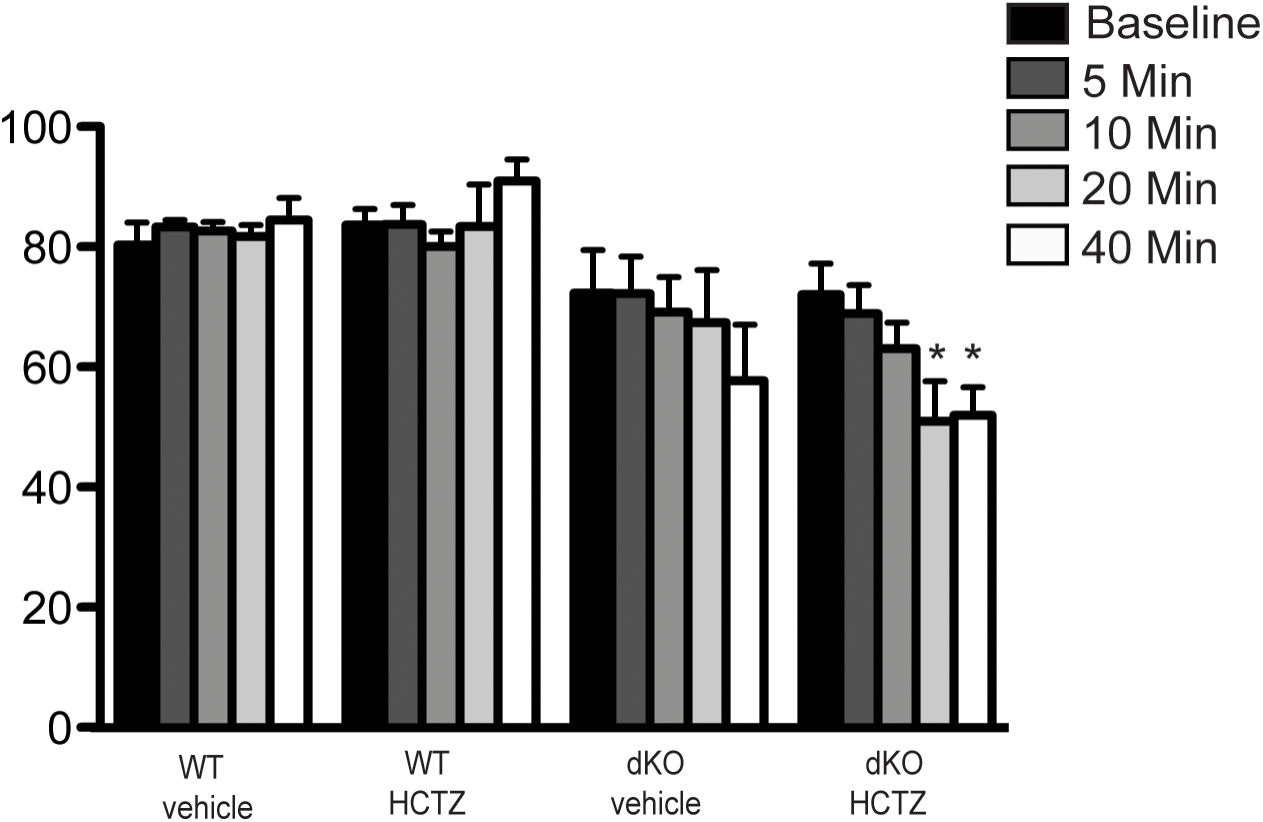
Intra-arterial BP monitoring in anesthetized WT and pendrin/NCC dKO mice before and after HCTZ. Mean arterial BP was measured at baseline and after HCTZ injection in WT (n = 4) and pendrin/NCC dKO mice (n = 5) at 5, 10, 20 and 40 minutes. One-way repeated measures ANOVA was performed; * P< 0.05 vs baseline.

To confirm the reduction in the BP of pendrin/NCC dKO mice and determine the magnitude and onset of the hypotensive action of HCTZ, intra-arterial BP was monitored in anesthetized mice according to the Methods. As indicated in Fig 3, the arterial BP reduced significantly in pendrin/NCC dKO mice as early as 20 minutes and remained low at 40 minutes after HCTZ. The vehicle treatment (propylene glycol/ethyl alcohol in 4/1 ratio) did not significantly affect the arterial BP in WT or pendrin/NCC dKO mice compared to baseline levels. An insignificant BP reduction in the volume depleted pendrin/NCC dKO mice treated with vehicle was observed over time compared to WT mice, likely due to the impact of anesthesia in volume depleted animals.

### Effect of HCTZ on the cardiac output

**Fig 4:**
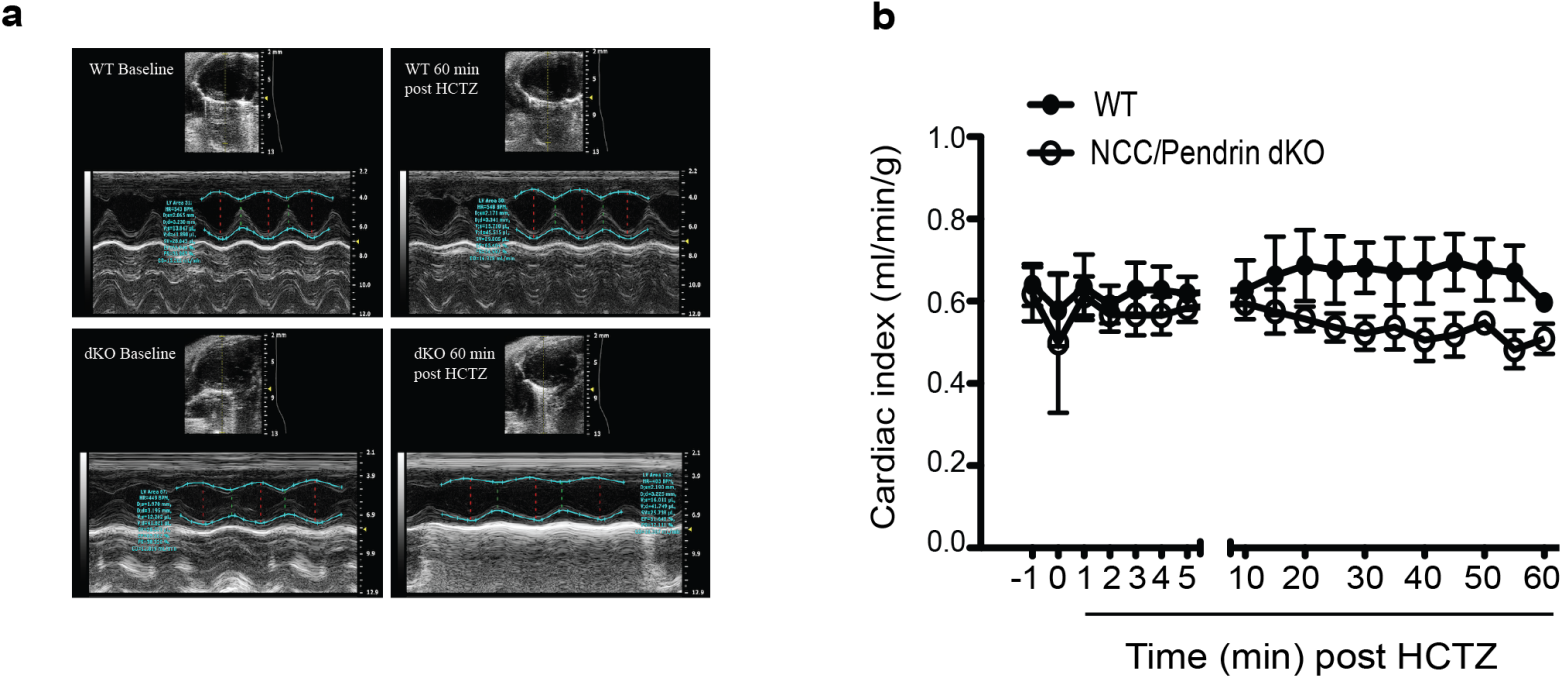
Echocardiography in anesthetized WT and pendrin/NCC dKO mice before and after HCTZ. (a) M-mode images of cardiac function of WT and pendrin/NCC dKO mice before and after HCTZ. (b) The examination of cardiac index as measured by echocardiography does not demonstrate any significant reduction in cardiac output of pendrin/NCC dKO mice after HCTZ treatment (n = 4 each group).

To ascertain the mechanism of HCTZ-induced hypotension in pendrin/NCC dKO mice, cardiac function was examined in anesthetized WT and dKO mice using high frequency echocardiography. Fig 4A shows a representative echocardiogram from WT and pendrin/NCC dKO mice at baseline and 60 minutes after HCTZ treatment. Our results demonstrate that cardiac index (ml/min/gram body weight) in pendrin/NCC dKO and WT mice were comparable before and after HCTZ (Fig 4B). The absence of any major reduction in cardiac index in the presence of a significant reduction in systemic BP (Figs 2A and 3) strongly suggest that the hypotensive effect of HCTZ in pendrin/NCC dKO mice is independent of changes in cardiac output. While there is a minor trend toward decreased function in pendrin/NCC dKO mice, this reduction does not explain the robust drop in BP of pendrin/NCC dKO mice treated with HCTZ.

### Effect of HCTZ on BP in NCC KO mice on a salt restricted diet

**Fig 5:**
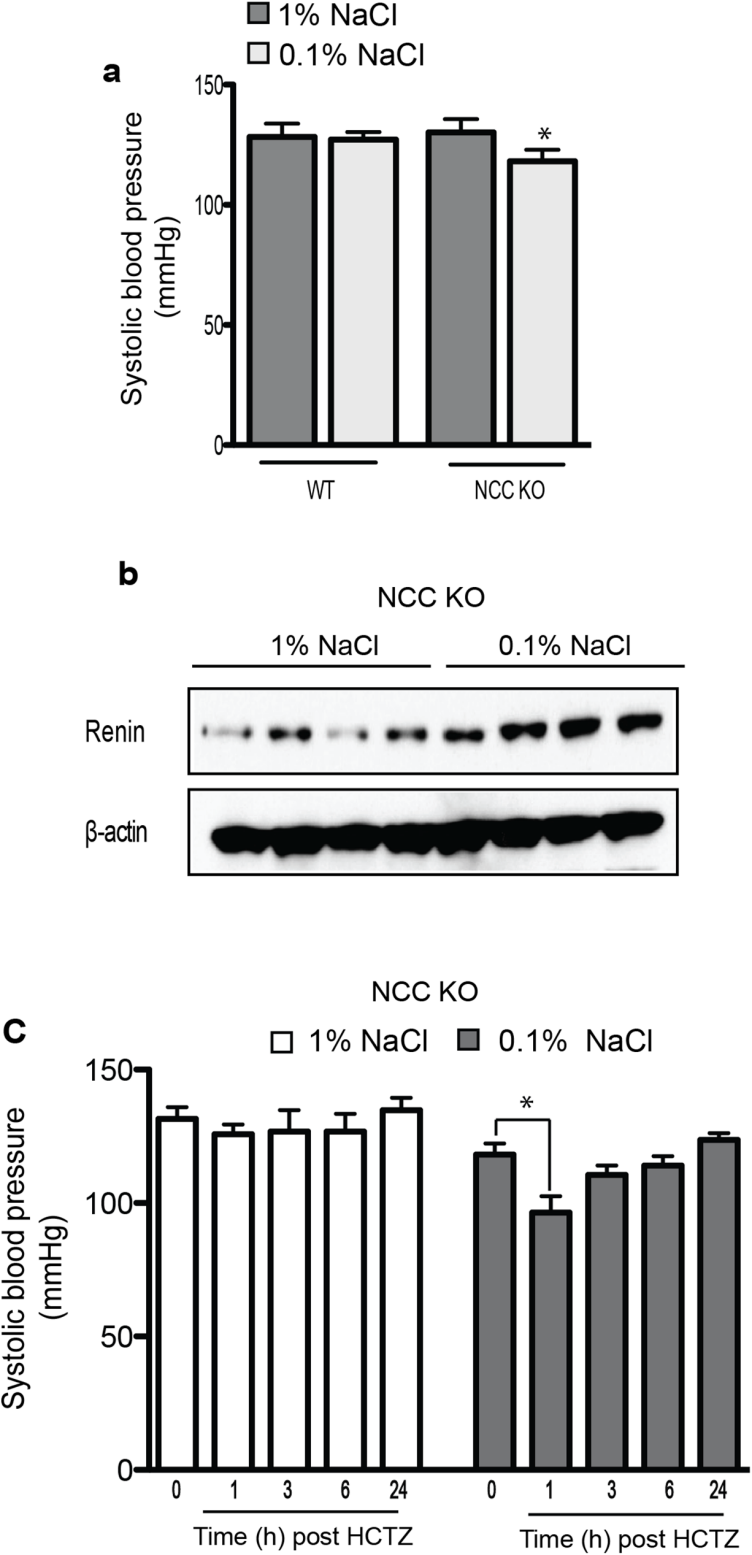
Effect of HCTZ on systemic BP of NCC KO mice on a salt restricted diet. NCC KO mice were placed on low salt (0.1% NaCl) diet for 14 days and the effect of HCTZ on the systolic BP of was examined. (a) The baseline systolic BP is significantly lower in NCC KO mice fed low salt (0.1% NaCl) diet for two weeks compared to similarly treated WT. (b) Western blot analysis shows renin protein expression is increased in NCC KO mice placed on a 0.1% NaCl diet. (c) HCTZ lowers BP in NCC KO mice on low salt diet but not their littermate in regular diet. (n = 4 each group); * P< 0.05 vs baseline.

Unlike NCC KO mice, pendrin/NCC dKO mice are severely volume depleted subsequent to salt wasting and volume depletion (Fig 1). Therefore, mice with single deletion of NCC (NCC KO mice) were subjected to salt restriction (0.1% NaCl) for 2 weeks, a maneuver that develops mild intravascular depletion and reduces the baseline BP [13]. Fig 5A and 5B shows that reduced dietary salt intake (0.1% NaCl) for 2 weeks reduces the systemic BP and induces the intravascular volume reduction representing by enhanced renin expression in kidneys of NCC KO compared to those on normal salt diet (1% NaCl). Further, we test the effects of HCTZ in the BP of NCC KO mice on salt deplete diet *vs*. normal salt diet. As shown in Fig 5C, HCTZ significantly decreases BP in salt depleted NCC KO mice. These results contrast with those in NCC KO mice on a normal salt diet that did not show any reduction in their BP in response to HCTZ (Fig 5C).

### Role of BK channels in the extra renal hypotensive effects of HCTZ

**Fig 6:**
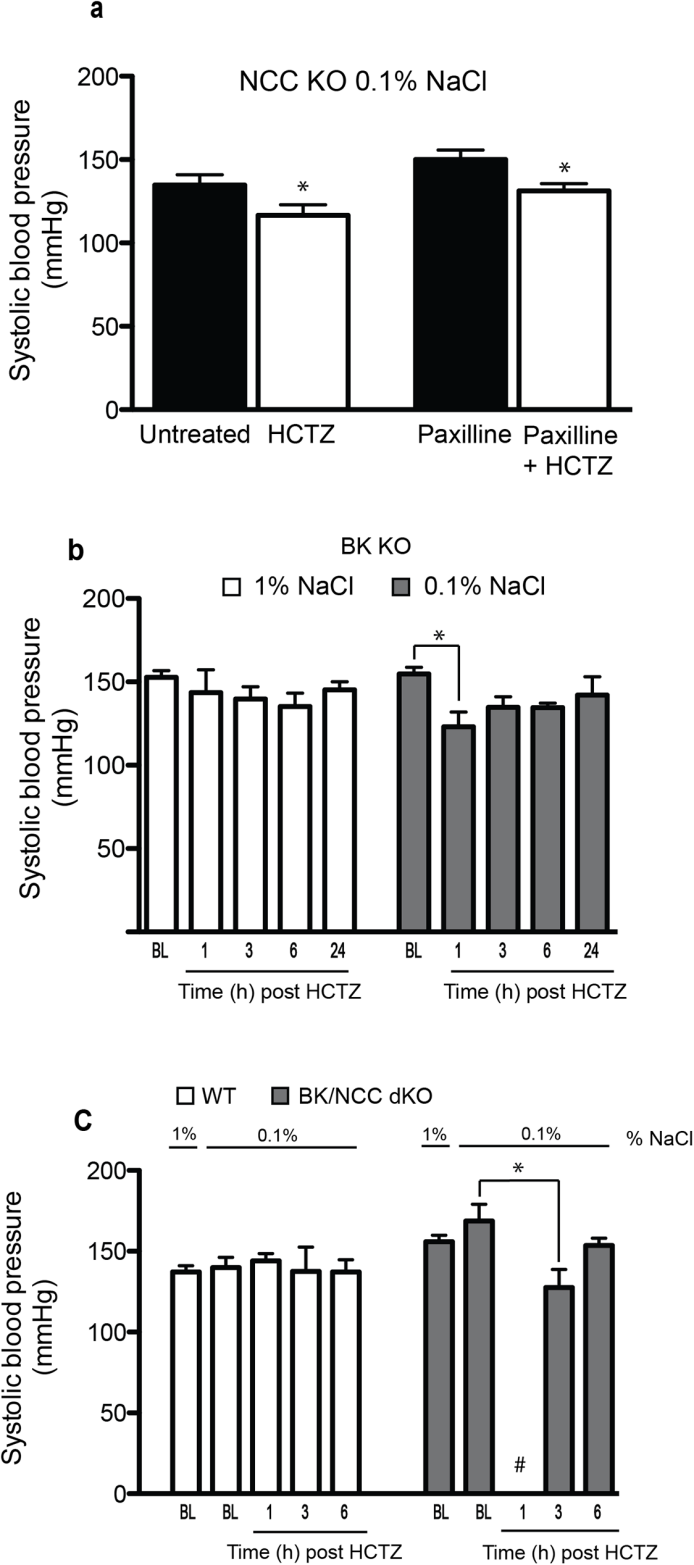
Role of BK channels in the effects of HCTZ on systemic BP. (a) Salt restricted NCC KO mice (1% NaCl, 2 weeks) were treated with HCTZ in presence of 8mg/kg of paxilline, paxilline failed to prevent the lowering BP effects at 1 hour after HCTZ; * P< 0.05. (b) HCTZ effects in BK KO mice. HCTZ lowers the BP of BK KO mice on 0.1% NaCl where it did not change their BP on normal diet (1% NaCl) (n = 4 each group). (c) HCTZ reduces the BP of BK/NCC dKO mice on 0.1% NaCl diet compared to the same treated WT mice (WT n = 4 vs BK/NCC dKO n = 6).

Calcium activated potassium channels and particularly the large conductance (BK) channels in vascular smooth muscle cells (VSMC) have been postulated as potential extra renal targets of HCTZ [6, 7, 12]. BK channel activation in VSMC may play an important role in systemic BP regulation through vasodilation [27–30]. Published reports indicate that BK channel deletion impairs vasodilation and results in elevated systemic blood pressure [31]. To ascertain the role of BK channels as mediators of HCTZ hypotensive effects, wild type and NCC KO mice on a low salt diet were treated with HCTZ in the presence or absence of paxilline, a specific BK channel inhibitor [32]. Fig 6A demonstrates that pretreatment with paxilline (8 mg/kg *i*.*p*) did not abrogate the hypotensive effects of HCTZ in salt restricted NCC KO mice, as shown by comparable reduction in systemic blood pressure in the presence or absence of paxilline.

To further investigate the role of BK channels in mediating the hypotensive effects of HCTZ, mice lacking the alpha subunit of BK channels (BK KO) were used. The results indicate that HCTZ significantly reduced the systemic blood pressure in BK KO mice on a low salt (0.1% NaCl) *vs* normal (1% NaCl) diet for two weeks (Fig 6B). HCTZ had no effect on BP of BK KO mice on a normal diet (Fig 6B). To probe this issue further, BK/NCC double KO mice were generated.

At baseline, BK/NCC dKO mice display significant enhancement in the expression of renin in their kidneys vs. WT, NCC KO and BK KO mice (S3A Fig). The expression of the collecting duct salt and water channels (ENaC and AQP-2) were examined next. As indicated, the NCC/BK dKO mice exhibited the upregulation of cleaved form (70 kDa) of ENaC γ subunit in their kidneys vs. BK KO or WT mice (S3A Fig). NCC KO mice showed a similar pattern (S3A Fig). The expression of kidney AQP2 is mildly reduced in BK KO mice vs. WT mice, but is comparable in kidneys of WT and BK/NCC dKO mice (S3B Fig). The exact mechanism of renin activation in kidneys of BK/NCC dKO mice (S3A Fig) remains speculative at the present.

To test the hypotensive effects of HCTZ in this new model, BK/NCC dKO mice were subjected to a low salt diet for 2 weeks (similar to NCC KO mice in Fig 5) and then treated with HCTZ. Fig 6C shows that HCTZ reduced the BP of salt restricted BK/NCC dKO but not WT mice at 1 hr and 3hrs post treatment.

### Vascular reactivity in response to HCTZ

**Fig 7:**
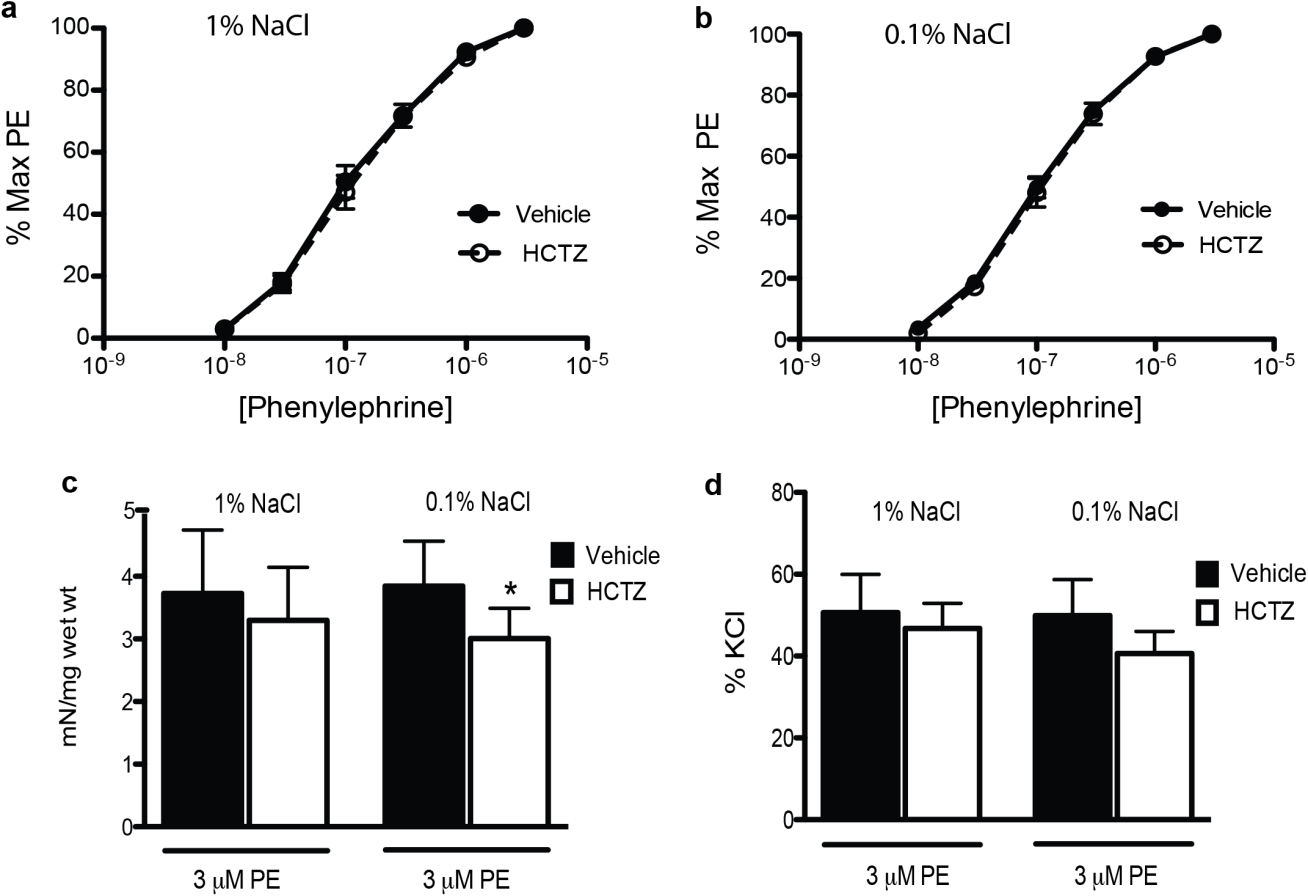
Effect of HCTZ on phenylephrine contraction of aorta in vitro from NCC KO mice with salt restricted (0.1%) and normal diet (1%). Cumulative concentration-contraction curves were elicited in aorta segments exposed to 100 μM hydrochlorothiazide (HCTZ) or DMSO (0.1%, vehicle control) from NCC KO mice with normal salt (a) and salt restricted diet (b). Contraction was calculated as mN/mg wet weight (c), and as percent of 50 mM KCl (d); * P< 0.05; n = 5 each group.

To test whether HCTZ has a direct vasorelaxtion effects in the blood vessels, the aorta reactivity in response to HCTZ experiments were performed. The contractile sensitivity to phenylephrine of aorta exposed to HCTZ and DMSO from NCC KO mice exposed to normal (1% salt) and restricted diet (0.1% salt) was also not different as demonstrated by the overlapping concentration-contraction response curves calculated as percent 3 μM phenylephrine contraction (Fig 7A and 7B). In aorta from NCC KO mouse with normal salt diet, maximal contraction to phenylephrine in the presence of 100 μM HCTZ and 0.1% DMSO (vehicle) was not significantly different as expressed both as a percent of the 50 mM KCl contraction as well as mN/mg wet weight (Fig 7B).

In aorta from NCC KO mouse subjected to salt restricted diet, 100 μM HCTZ decreased maximal contraction to 3 μM phenylephrine when expressed as mN/mg wet weight (p<0.04, Student’s paired t-test and p>0.05, Student’s unpaired t-test). In addition, the reduction of maximal contraction to 3 μM phenylephrine did not achieve significance when expressed as percent of 50 mM KCl (p<0.08, Student’s paired t-test). Contractile sensitivity to phenylephrine was also not altered by 100 μM HCTZ (Fig 7A and 7C).

## Discussion

The mechanisms of BP reduction by thiazide derivatives including HCTZ are not fully understood. Despite the fact that HCTZ shows superior hypotensive effects compared to other potent diuretics, which implies the non-diuretic mechanisms, there are no *in vivo* studies distinguishing between diuretic and non-diuretic hypotensive effects of HCTZ. The uncertainty about HCTZ hypotensive mechanisms is likely due to 1) both mechanisms may be operating simultaneously and 2) the paucity of knowledge of pathophysiological circumstances that determine by which mechanism HCTZ reduces the BP. In this study, we were able to uncover conditions by which HCTZ is able to reduce the BP via non-diuretics (no enhanced diuresis) mechanisms.

In the kidney, the sodium chloride co-transporter (NCC, gene bank designation SLC12A3) is the main target of thiazide derivatives. The NCC is exclusively expressed on the apical membrane of the DCT in mammalians [2–4]. Loss of function mutations in NCC causes Gitelman Syndrome (GS), a condition manifested with magnesium wasting, hypokalemia and metabolic alkalosis [33, 34]. Systemic BP is reduced by approximately 7–8 mm Hg in patients with Gitelman Syndrome, which is similar to the results seen in response to thiazide treatment [35]. Published reports indicate that pendrin inactivation enhances the diuretic action of HCTZ, as shown by enhanced salt and water excretion in response to HCTZ [9, 10]. These results indicate the compensatory role of pendrin in salt reabsorption in the presence of NCC inhibition by thiazides.

In the present studies we used animals with specific deletion of NCC in order to ascertain the non-diuretic effect of HCTZ on BP. Our results demonstrated that in NCC KO mice on normal 1% salt intake, HCTZ had no discernible effect on BP (Fig 5). However, HCTZ was able to reduce the systemic BP in salt restricted (0.1% salt) NCC KO mice (Fig 5), The most dramatic reduction in BP by HCTZ occurred in pendrin/NCC dKO mice (Figs 2 and 3). The pendrin/NCC dKO mice display significant systemic vasoconstriction, as determined by a robust hypotensive response to angiotensin receptor blocker (Fig 1). Correction of volume depletion and suppression of renin/angiotensin pathway by salt replacement blunted the hypotensive effect of HCTZ in pendrin/NCC dKO mice. Echocardiography results confirmed that HCTZ did not reduce the BP through reduction of cardiac function. There is a slight but insignificant reduction in cardiac output at the end of experiment after HCTZ injection in pendrin/NCC dKO mice. However, the observed significant reduction in BP of pendrin/NCC dKO mice in response to HCTZ (Fig 3) cannot be explained by a minor reduction in cardiac function, indicating that the reduction in vascular resistance is the main cause of hypotension by HCTZ. Taken together, it is likely that the hypotensive effect of HCTZ is through the reduction in systemic vascular resistance.

In addition to inhibiting NCC in the kidney, thiazide derivatives have shown the capability to cause vasodilation [6, 7, 12]. While the preponderance of evidence points to kidney as the predominant site of its action, the exact contribution of natriuresis (renal) vs. vasodilation (extra renal) to the control of BP by thiazides remains speculative. It is however worth mentioning that the prevailing view is that thiazides lower BP predominantly via enhanced salt excretion [4, 36].

The majority of published reports examining the effect of HCTZ on BP have shown inconsistent or conflicting results, primarily due to age, status of vascular resistance or the presence of underlying kidney disease in treated subjects. For example, HCTZ in elderly patients with high systemic vascular resistance caused significant hypotension without causing a change in cardiac output [37]. It was also found that HCTZ reduces the BP in these patients directly by reducing the vascular resistance. These results were contradicted in a separate study that showed the HCTZ-induced reduction in BP was associated with a contraction in plasma volume, consistent with enhanced salt excretion secondary to the inhibition of NCC in the kidney [4]. Studies by Van Brummelen et al showed that subjects who responded to HCTZ (more than 10% reduction in BP) had lower peripheral vascular resistance compared to non-responders (less than 10% reduction in BP) [5]. A study on the direct effect of HCTZ on blood vessels in patients with Gitelman Syndrome, who lack a functional NCC, concluded that NCC does not play an important role in the vasodilator action of HCTZ [7]. Collectively, these results point to the presence of extra renal action of HCTZ; however, it is not clear whether or not the initial inhibition of NCC in the kidney plays any role in modulating the activity of the extra renal targets of HCTZ.

Calcium activated potassium channels (KCa) and particularly the large conductance (BK) channels in VSMCs have been known as targets of HCTZ [6, 7, 12]. BK is the only KCa channel expressed in VSMCs, and plays a major role in the regulation of vascular tone [38, 39]. These channels are activated by increases in [Ca2+]i and membrane depolarization, and play an important role in modulating the vasoconstriction [27, 28, 40]. Iberiotoxin, tetraethyl ammonium (TEA), and paxilline are inhibitors of BK channels and lead to membrane depolarization and vasoconstriction [40, 41]. To address the role of BK channels in the hypotensive effects of HCTZ, we demonstrated that BK channels blockers (paxilline) did not abolish the hypotensive effects of HCTZ in restricted salt diet NCC KO mice. Further, HCTZ reduces the BP in BK KO and BK/NCC dKO mice on salt restriction diet but not WT mice (Fig 6). These results strongly suggest that BK channels do not mediate the extra renal effect of HCTZ on systemic BP.

Consistent with the lack of role for BK channels in HCTZ reduction of arterial pressure, the overall minimal inhibitory effect of 100 μM HCTZ on phenylephrine contraction in aorta *in vitro* suggests that the lowered arterial pressure by HCTZ was independent of a direct action of HCTZ on the vasculature. Notably, the concentration of HCTZ was employed in previous in *vitro* vascular studies [42, 43].

Although an explanation for the ability of HCTZ to inhibit in vitro contraction in other studies may reflect differences in the vascular preparation, this explanation is clearly not relevant to the present findings because HCTZ decreased arterial pressure in NCC KO with salt restricted diet and pendrin/NCC dKO. On the other hand, the absence of the direct action of HCTZ in the blood vessels might be due the lack of *in vivo* plasma circulating factor(s) induced by volume depletion that is (are) not existing in *ex vivo* system. Further, the effects of HCTZ on contraction need to be investigated in resistance-type vessels.

Our results indicate that in animals with increased renin angiotensin levels and systemic vasoconstriction, HCTZ reduces the BP independent of NCC inhibition and without causing an increase in salt excretion. These results indicate that HCTZ can lower systemic BP through extra renal mechanisms only in the setting of systemic vasoconstriction. Published studies indicate that HCTZ and salt restriction synergistically reduce the systemic BP in hypertensive individuals [44, 45]; however, the mechanism of this effect remains speculative.

The Slc4a8 which mediates sodium dependent chloride bicarbonate exchange (NDCBE) has been proposed to be expressed in B-intercalated cells of CCD and inhibited by HCTZ [19]. The absence of increased urine volume or salt excretion in NCC KO mice or pendrin/NCC dKO mice by HCTZ (Results) excludes an important role for NDCBE in compensatory salt absorption in these animals. A recent study indicated that HCTZ inhibits angiotensin II type one receptor (AT1) pathway in the ischemic heart [46]. It is plausible that the hypotensive effect of HCTZ may be mediated in part via AT1 receptor in vascular smooth muscle cells.

Our studies demonstrate that HCTZ has a robust hypotensive effect in mice lacking NCC, primarily in the setting of increased systemic vascular resistance. This effect is not mediated through direct vasodilation and the calcium activated K^+^ (BK) channels. Whether HCTZ reduces the BP through direct or indirect actions on the small resistance blood vessels need to be further investigated. We propose that patients with increased systemic vascular resistance secondary to increased angiotensin II, such as those with systolic heart failure, liver cirrhosis or volume depletion may be at increased risk of systemic hypotension when taking thiazide derivatives.

## Supporting information

S1 Fig A representative tail DNA genotyping in WT, NCC KO, BK KO, and BK/NCC dKO mice. Using gene specific PCR primers tail DNA genotyping was performed to identify BK KO, NCC KO and BK/NCC dKO mice (see Methods for detail).
(TIF)

S2 Fig Balance studies in WT and pendrin/NCC dKO mice at baseline and after HCTZ treatment. Metabolic balance studies showed that 3 days of (40mg/kg) HCTZ treatment causes an unexpected reduction in the urine output (a), water intake (b) and food intake (c) of pendrin/NCC dKO mice compared to their WT littermates. (n = 4 each group); ** P<0.01; *** P<0.001 for Baseline vs. HCTZ treatment.
(TIF)

S3 Fig Kidney expression of renin, ENaC, and AQP2 in WT, NCC KO, BK KO, and BK/NCC dKO mice. (A) (top panel) Western blot analysis using renin antibodies indicates a significant increase in renin expression in kidneys of BK/NCC dKO mice compared to NCC KO, BK KO or WT mice. (middle panel) Western blot analysis using ENaC gamma subunit antibodies indicates a significant increase in the expression of cleaved ENaC gamma subunit band in kidneys of BK/NCC dKO mice and NCC KO mice but not in BK KO or WT mice. (bottom panel) Expression of B-actin as a marker of protein loading. (B) Western blot analysis using AQP-2 antibodies indicates a reduction in AQP2 expression in kidneys of BK KO mice compared to other groups. The expression of AQP-2 was comparable in kidneys of WT and BK/NCC dKO mice. Expression of B-actin as a marker of protein loading is shown at the bottom.
(TIF)
