# Supplementary figures and images for: The non-diuretic hypotensive effects of thiazides are enhanced during volume depletion states
Source: PLoS One. 2017 Jul 18;12(7):e0181376. doi: 10.1371/journal.pone.0181376 (PMC5515454; doi:10.1371/journal.pone.0181376)

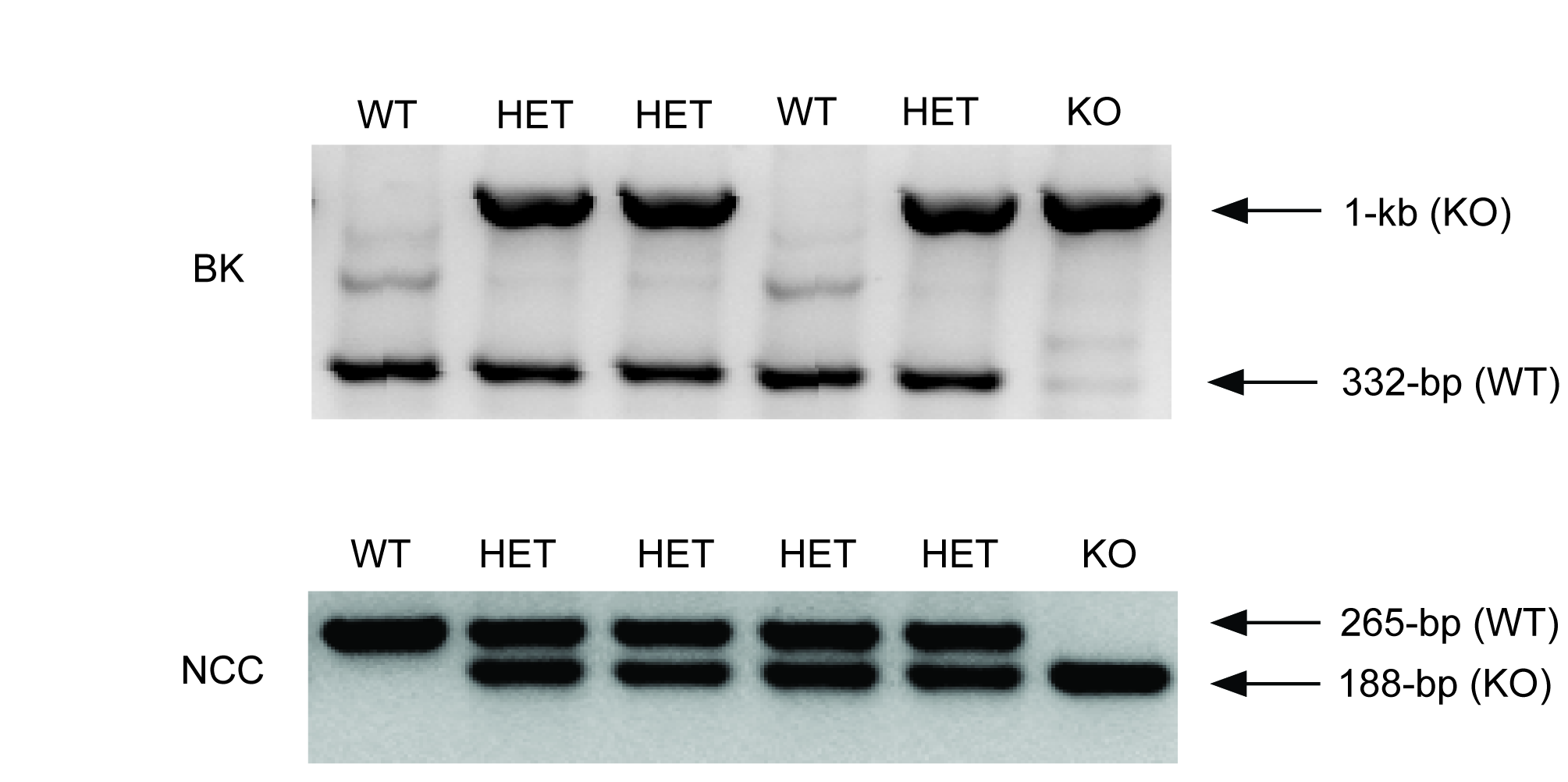

Supplement: S1 Fig — Using gene specific PCR primers tail DNA genotyping was performed to identify BK KO, NCC KO and BK/NCC dKO mice (see Methods for detail). (TIF) [file pone.0181376.s001.tif]

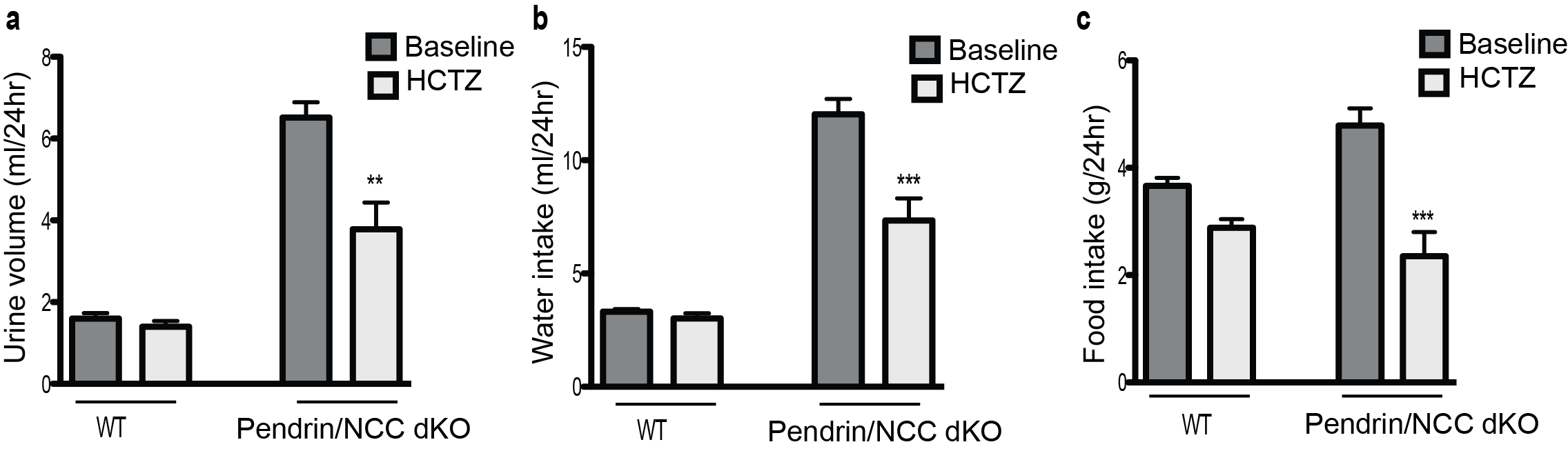

Supplement: S2 Fig — Metabolic balance studies showed that 3 days of (40mg/kg) HCTZ treatment causes an unexpected reduction in the urine output (a), water intake (b) and food intake (c) of pendrin/NCC dKO mice compared to their WT littermates. (n = 4 each group); ** P<0.01; *** P<0.001 for Baseline vs. HCTZ treatment. (TIF) [file pone.0181376.s002.tif]

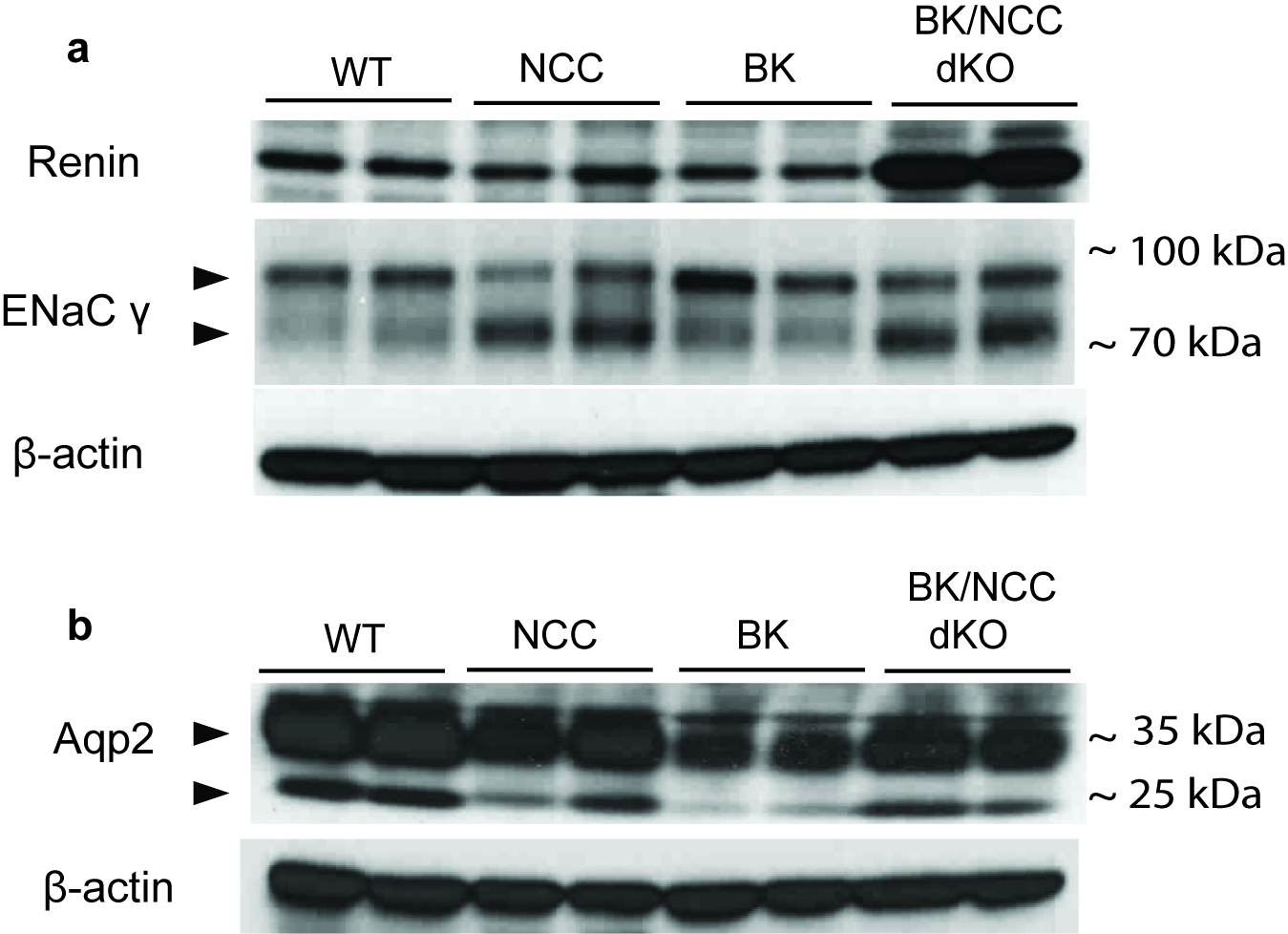

Supplement: S3 Fig — (A) (top panel) Western blot analysis using renin antibodies indicates a significant increase in renin expression in kidneys of BK/NCC dKO mice compared to NCC KO, BK KO or WT mice. (middle panel) Western blot analysis using ENaC gamma subunit antibodies indicates a significant increase in the expression of cleaved ENaC gamma subunit band in kidneys of BK/NCC dKO mice and NCC KO mice but not in BK KO or WT mice. (bottom panel) Expression of B-actin as a marker of protein loading. (B) Western blot analysis using AQP-2 antibodies indicates a reduction in AQP2 expression in kidneys of BK KO mice compared to other groups. The expression of AQP-2 was comparable in kidneys of WT and BK/NCC dKO mice. Expression of B-actin as a marker of protein loading is shown at the bottom. (TIF) [file pone.0181376.s003.tif]
